# Redox Metabolism in Ecophysiology and Evolution

**DOI:** 10.3390/antiox12091769

**Published:** 2023-09-16

**Authors:** Daniel C. Moreira, Tania Zenteno-Savín, Marcelo Hermes-Lima

**Affiliations:** 1 Research Center in Morphology and Applied Immunology, Faculty of Medicine, University of Brasilia, Brasilia 70910-900, Brazil; 2 Centro de Investigaciones Biológicas del Noroeste, La Paz 23096, Mexico; tzenteno04@cibnor.mx; 3 Department of Cell Biology, University of Brasilia, Brasilia 70910-900, Brazil; hermes@unb.br

Aerobic organisms have developed a complex system of endogenous antioxidants to manage the reactivity of oxygen and its byproducts. This interplay between reactive oxygen/nitrogen species (RONS) and defensive mechanisms has profound implications, extending well beyond mere interactions of attack and defense. Redox metabolism, encompassing non-radical redox metabolites (e.g., hydrogen peroxide and glutathione), redox-sensitive transcription factors (e.g., Nrf2 and FOXOs), and redox-sensitive proteins (e.g., thioredoxins and peroxiredoxins), forms a network of signaling pathways with extensive effects on various processes in aerobic organisms, spanning from circadian rhythms to the regulation of lifespan.

Oxidative stress, stemming from an imbalance between RONS production and cellular antioxidant capacity, is now recognized as a pivotal determinant of the life history of organisms. Environmental stressors have the potential to disrupt the equilibrium of redox reactions, eliciting compensatory adaptive responses. Modulations in redox metabolism have been documented in a wide range of species, spanning various phylogenetic groups and exposed to diverse environmental stressors such as fluctuations in temperature, changes in water availability, shifts in oxygen levels, exposure to UV radiation, pollutants, and more.

This Special Issue is dedicated to investigating the responses of redox metabolism in organisms experiencing alterations in individual or combined environmental factors, whether of biotic or abiotic origin. Comprehending these responses to stress inducers is critical for unraveling the role of oxidative stress in the survival and fitness of organisms. By examining how organisms confront the challenges posed by these changing factors, valuable insights can be gained into the interplay between redox metabolism and the overall health of ecosystems. This research not only enhances our comprehension of the underlying mechanisms but also illuminates potential strategies for mitigating the repercussions of environmental changes on biodiversity and ecological stability.

Arango et al. [1] investigated possible physiological costs associated with olive ridley sea turtles’ nesting behavior, either solitary or in "arribadas" (mass aggregations). The study examined metabolic differences between the nesting modes and found that arribada nesters were larger and had higher thyroid hormone levels. Moreover, turtles that presented arribada behavior had metabolic pathways related to phospholipid and amino acid metabolism, as well as catabolic processes, upregulated compared with those reproducing solitarily. However, arribada nesters exhibited higher levels of oxidative damage in terms of lipid peroxidation and protein oxidation, suggesting a trade-off between fitness benefits and oxidative stress associated with arribada nesting.

Cassier-Chauvat et al. [2] examined the significance of the glutathione (GSH) system in defending cells against various stressors across phylogenetically diverse organisms. They underscored the crucial roles of glutathione-dependent systems in maintaining redox balance, detoxifying harmful substances, and regulating iron metabolism. The study delved into multiple pathways, including GSH synthesis, degradation, recycling, conjugation, glutathionylation/deglutathionylation, and iron–sulfur cluster [Fe-S] synthesis and repair. The authors ultimately suggested that cyanobacteria, regarded as the originators of the GSH system, are the ideal model organisms for further research into its components.

Cubillos et al. [3] investigated the impact of experimental radiation exposure on the burrowing behavior and oxidative damage in *Anthopleura hermaphroditica*, an intertidal anemone. They exposed adults and juveniles to different radiation treatments, including photosynthetically active radiation (PAR), ultraviolet A radiation (UVA), and ultraviolet B radiation (UVB), both with and without sediment. Animals were exposed either to PAR alone, PAR + UVA, or PAR + UVA + UVB. The results showed that exposure to PAR + UVA + UVB radiation led to faster burrowing responses in both adults and juveniles compared to PAR alone. Juveniles had higher basal levels of oxidative stress markers (protein carbonyl and malondialdehyde) than those in adults. Exposure to PAR + UVA + UVB further increased oxidative damage to protein and lipids in juveniles, regardless of the presence or not of sediment. However, the presence of sediment protected adults against various radiation treatments. The research found that *A. hermaphroditica* burrows into sediment as a behavioral response to UVR radiation, indicating their evasion of UVB radiation and UVR-induced oxidative stress.

Ferreira-Cravo et al. [4] explored the role of glutathione (GSH) in managing redox homeostasis during anoxia and reoxygenation in *Helix aspersa*, a model for anoxia tolerance. The researchers depleted the total GSH pool inhibiting GSH synthesis with L-buthionine-(S, R)-sulfoximine (BSO) and subjected snails to anoxia. BSO alone led to GSH depletion but no other significant changes in antioxidants and oxidative stress markers, while anoxia triggered an increase in glutathione peroxidase (GPX) activity. However, GSH depletion before anoxia raised the GSSG/tGSH ratio during anoxia, indicating a disrupted redox balance. Therefore, GSH depletion does not directly induce oxidative stress in snails but impairs their ability to manage oxidative challenges during anoxia. The study indicates the importance of GSH in countering oxidative challenges during hypoxia and reoxygenation in land snails. 

Jacobs et al. [5] investigated the impact of aridification on wild common mole rats and their oxidative status along an aridity gradient. Liver oxidative status showed no significant change with aridity levels. However, aridity did affect the total antioxidant capacity (TAC) and oxidative stress index (OSI) of the kidney, with the most arid habitats showing the highest TAC. These findings were interpreted in the context of the kidney’s role in water balance and retention. The authors conclude that social common mole rats in arid environments prioritize and protect their kidneys with higher antioxidants, mitigating the impact of aridification on oxidative stress. Nevertheless, more research is needed to further explore the effects of aridity on mammalian oxidative ecology, particularly in the context of climate change.

Malagoli et al. [6] reviewed the critical role of reactive oxygen species (ROS) in mollusks’ physiological functions and their relevance in both environmental adaptation and immune responses. The authors focused on highly invasive mollusks (e.g., *Mytilus galloprovincialis*, *Dreissena polymorpha*, *Achatina fulica*, and *Pomacea canaliculata*) and their ability to manage ROS production during challenging conditions. The review emphasizes that the capacity to maintain redox homeostasis under oxidative pressure can be advantageous when facing environmental and immunological challenges, potentially making it a trait associated with invasiveness. 

Ramirez et al. [7] investigated the effect of lipoic acid, a mitochondrial coenzyme that can act as an antioxidant or pro-oxidant, on energy metabolism and redox balance. Using *Artemia* sp. nauplii as a model organism, the authors found that lipoic acid treatment positively influenced the activity of the electron transport system, particularly at higher concentrations and longer exposure times. Additionally, lipoic acid promoted glucose catabolism, decreased ROS production, and influenced protein levels and total ammoniacal nitrogen production under specific conditions.

Yang et al. [8] investigated the response of *Cherax destructor*, a freshwater crustacean, to different temperatures. After 8 weeks of exposure to 30 °C, 25 °C, 20 °C, 15 °C, or 10 °C, levels of components of the glutathione redox system (GSH, GSSG, GST, and GR) and transcript levels of proteins that respond to temperature stress (heat-shock proteins and cold shock-domain proteins) were quantified in the hepatopancreas. With a focus on the effect of low temperatures, the study showed that growth indicators such as weight gain and length gain decreased at lower temperatures. Exposure to 10 °C also decreased GST activity and GSSG/GSH ratio, while increasing GSH levels in hepatopancreas when compared with animals maintained at 25 °C. Different temperatures modulated the expression of temperature stress-responsive proteins, among which HSP60, HSP70, and CSP were upregulated at 10 °C. Transcriptome sequencing identified differentially expressed genes related to endocrine disorders, glucose metabolism, antioxidant defense, and immune responses. The findings suggest that low temperature inhibited basal metabolism and immune ability but increased redox buffering capacity and temperature–stress response in the crayfish.

Zhang et al. [9] focused on the effects of mercury (as HgCl_2_) toxicity on *Procambarus clarkii*, a type of crayfish commonly found in aquatic environments worldwide. The study examined the acute impact of Hg on various aspects, including biochemical responses, histopathology, hepatopancreatic transcriptome, and intestinal microbiome. Hg exposure led to significant changes in redox metabolism markers, including increases in malondialdehyde (MDA) content and a general trend of decreasing antioxidant levels. Structural damage was observed in the hepatopancreas and intestines. RNA-seq identified differentially expressed genes (DEGs) mainly associated with redox metabolism, ion transport, drug metabolism, immune response, and apoptosis. The study also showed that Hg exposure altered the composition of the intestinal microbiome. The findings provide insights into the mechanisms of Hg-induced toxicity in aquatic crustaceans at various levels, including tissue, cellular, molecular, and microbial levels.

Jacobs et al. [10] explored several factors that could be associated with redox balance and oxidative stress, such as phylogeny, social behavior, captivity, environmental aridity, resting metabolic rate (RMR), and maximum lifespan potential (MLSP). 

The authors found that higher levels of oxidative markers in plasma are associated with higher MLSP by comparing several species of African naked mole rats. Such association was more evident in social species than in solitary species. Additionally, oxidative stress marker levels decrease with a higher aridity index, and wild-caught mole rats show higher antioxidant levels. The concept of hormesis, which involves a biphasic response to stimuli, is proposed as a potential mechanism contributing to longevity in certain members of the Bathyergidae family.

These studies provide further evidence of the role of oxidative stress as a key factor in the life history of living organisms. Changes in RONS production and antioxidant defenses in response to different biotic and abiotic factors (individually and/or combined) contribute to regulating signaling pathways that dictate whether an aerobic species will survive. Studies such as that of Jacobs et al. [5] highlight the role of combined responses at biochemical, physiologic, behavioral, and/or population levels in responding to those factors. Further, the studies included in this Special Issue suggest compensatory adaptive responses may modulate redox metabolism in phylogenetically diverse species. Closer analyses of these results will provide clues to understanding the evolution of antioxidant defense systems, the underlying mechanisms, the subtle connections of the redox metabolism with other vital systems, such as the immune system, and its participation in shaping existent biodiversity. Results from studies of redox metabolism with a comparative focus can provide clues towards dealing with environmental and climate change, provide viable solutions for the conservation of species, and maintain the balance between environmental, animal, and human health in a sustainable manner. 

## References

[1] Arango B.G., Ensminger D.C., Moreno-Santillán D.D., Harfush-Meléndez M., López-Reyes E.M., Marmolejo-Valencia J.A., Merchant-Larios H., Crocker D.E., Vázquez-Medina J.P. (2022). Oxidative Stress Is a Potential Cost of Synchronous Nesting in Olive Ridley Sea Turtles. Antioxidants.

[2] Cassier-Chauvat C., Marceau F., Farci S., Ouchane S., Chauvat F. (2023). The Glutathione System: A Journey from Cyanobacteria to Higher Eukaryotes. Antioxidants.

[3] Cubillos V.M., Álvarez J.A., Ramírez E., Cruces E., Chaparro O.R., Montory J., Spano C.A. (2022). Effects of Ultraviolet Radiation on Sediment Burial Parameters and Photo-Oxidative Response of the Intertidal Anemone Anthopleura Hermaphroditica. Antioxidants.

[4] Ferreira-Cravo M., Moreira D.C., Hermes-Lima M. (2023). Glutathione Depletion Disrupts Redox Homeostasis in an Anoxia-Tolerant Invertebrate. Antioxidants.

[5] Jacobs P.J., Hart D.W., Merchant H.N., Janse Van Vuuren A.K., Faulkes C.G., Portugal S.J., Van Jaarsveld B., Bennett N.C. (2022). Tissue Oxidative Ecology along an Aridity Gradient in a Mammalian Subterranean Species. Antioxidants.

[6] Malagoli D., Franchi N., Sacchi S. (2023). The Eco-Immunological Relevance of the Anti-Oxidant Response in Invasive Molluscs. Antioxidants.

[7] Buitrago Ramírez J.R., Marreiro Gomes R.M., De Sousa Araujo A.C., Muñoz Buitrago S.A., Piraine Souza J., Monserrat J.M. (2023). The Effects of Lipoic Acid on Yolk Nutrient Utilization, Energy Metabolism, and Redox Balance over Time in *Artemia* sp.. Antioxidants.

[8] Yang Y., Xu W., Jiang Q., Ye Y., Tian J., Huang Y., Du X., Li Y., Zhao Y., Liu Z. (2022). Effects of Low Temperature on Antioxidant and Heat Shock Protein Expression Profiles and Transcriptomic Responses in Crayfish (Cherax Destructor). Antioxidants.

[9] Zhang L., Zhou Y., Song Z., Liang H., Zhong S., Yu Y., Liu T., Sha H., He L., Gan J. (2022). Mercury Induced Tissue Damage, Redox Metabolism, Ion Transport, Apoptosis, and Intestinal Microbiota Change in Red Swamp Crayfish (Procambarus Clarkii): Application of Multi-Omics Analysis in Risk Assessment of Hg. Antioxidants.

[10] Jacobs P.J., Hart D.W., Merchant H.N., Voigt C., Bennett N.C. (2023). The Evolution and Ecology of Oxidative and Antioxidant Status: A Comparative Approach in African Mole-Rats. Antioxidants.

